# Direct Composite Restorations on Permanent Teeth in the Anterior and Posterior Region – An Evidence-Based Clinical Practice Guideline – Part 1: Indications for Composite Restorations

**DOI:** 10.3290/j.jad.b5748881

**Published:** 2024-09-17

**Authors:** Diana Wolff, Cornelia Frese, Roland Frankenberger, Rainer Haak, Andreas Braun, Norbert Krämer, Gabriel Krastl, Falk Schwendicke, Esra Kosan, Eva Langowski, Caroline Sekundo, and Guideline Panel Members and Methodological Consultants

**Affiliations:** a Professor, Department of Conservative Dentistry, Heidelberg University, University Hospital Heidelberg, Germany. Supervision, funding acquisition, project administration, investigation, writing (review and editing).; b Adjunct Professor, Department of Conservative Dentistry, Heidelberg University, University Hospital Heidelberg, Germany. Project administration, investigation, writing (review and editing).; c Professor, Department of Operative Dentistry and Endodontology, University of Marburg, Germany. Project administration, investigation, writing (review and editing).; d Professor, Department of Cariology, Endodontology and Periodontology, University of Leipzig, Germany. Project administration, investigation, writing (review and editing).; e Professor, Department of Operative Dentistry, Periodontology and Preventive Dentistry, RWTH Aachen University, Germany. Investigation, writing (review and editing).; f Professor, Paediatric Dentistry, University of Gießen, Germany. Investigation, writing (review and editing).; g Professor, Department of Conservative Dentistry and Periodontology – Center of Dental Traumatology, University Hospital of Würzburg, Germany. Investigation, writing (review and editing).; h Professor, Department of Conservative Dentistry and Periodontology, LMU Klinikum, Germany. Project administration, investigation, writing (review and editing).; i Dentist, Department of Periodontology, Oral Medicine and Oral Surgery, Charité – Universitätsmedizin Berlin, Germany. Methodology, investigation, writing (review and editing).; j Dentist, Department of Conservative Dentistry, Heidelberg University, University Hospital Heidelberg, Germany. Methodology, investigation, writing (review and editing).; k Dentist, Department of Conservative Dentistry, Heidelberg University, University Hospital Heidelberg. Project administration, methodology, investigation, writing (original draft).

**Keywords:** adhesive restorations, composite resin, composite restorations, evidence-based medicine

## Abstract

**Purpose:**

This German S3 clinical practice guideline offers evidence-based recommendations for the use of composite materials in direct restorations of permanent teeth. Outcomes considered were the survival rates and restoration quality and process quality of the manufacturing process. Part 1 of this two-part presentation deals with the indication classes.

**Materials and Methods:**

A systematic literature search was conducted by two methodologists using MEDLINE and the Cochrane Library via the OVID platform, including studies up to December 2021. Six PICO questions were developed to guide the search. Recommendations were formulated by a panel of dental professionals from 20 national societies and organizations based on the collected evidence.

**Results:**

Composite materials are a viable option for the direct restoration of cavity Classes I–V and may also be used for restorations with cusp replacement, and tooth shape corrections. In the posterior region, direct composite restorations should be preferred over indirect composite inlays. For Class V restorations, composite materials can be used if adequate contamination control and adhesive technique are ensured.

**Conclusion:**

The guideline is the first to provide comprehensive evidence on the use of direct composite materials. However, further long-term clinical studies with comparators such as (modified) glass-ionomer cements are necessary. Regular updates will detail the future scope and limitations of direct composite restorations.

The burden of dental caries is substantial, with untreated adult caries being one of the most prevalent diseases globally, affecting nearly 30% of the population (29.4% [26.8–32.2%]) according to the Global Burden of Disease Study.^9^ Although preventive measures have reduced caries in industrialized nations like Germany, conservative dental treatments such as direct restorations and root canal treatments still comprise about 56% of all statutory dental services there, albeit with a declining trend.^7^ Various treatments are available for restoring carious tooth structure loss, repairing or replacing inadequate restorations, and restoring non-carious tooth structure loss. Cavities can be restored using direct restorative procedures or indirect workpieces. The decision path is based on the cavity class, the cavity size, extent and depth, the previous damage to the tooth, other local conditions such as the condition of the antagonist, patient factors such as cooperation, caries risk, prognosis and health policy aspects such as the insurance status.

The development of tooth-colored composite materials, a major advancement in dentistry, has facilitated minimally invasive treatment of tooth defects and cavities. Composites not only impress with their esthetic appearance, they also enable a much gentler approach to the design of primary cavities, excavation of carious lesions and secondary re-interventions due to strong adherence to dental hard tissues via adhesive techniques. The focus has shifted from “extension for prevention” to “prevention of extension,” utilizing modern materials and techniques for a minimally invasive treatment approach. Moreover, direct composite restorations are economically significant for healthcare systems. Over the past three decades, their use for treating caries-related and non-caries-related defects has grown. In 2021, 47.1 million direct restorations were performed in Germany for people with statutory health insurance, predominantly using direct composite materials.^34^ Direct restorative therapy thus comprises a substantial part of the dental care spectrum, and reliable data should be available on its indication, implementation and prognosis.

With the clinically demonstrable success of direct composite restorations in standard cavity Classes I to V, there has been an increasing expansion of indications over the last 20 years.^56^ Today, composite materials are used for extended and large cavities, for example with cusp replacements^33^ as well as for esthetic-functional corrections with regard to tooth position, shape and shade.^22,26^ This widespread use, even beyond the standard indications Class I to V, necessitates updated guidelines with strong evidence and recommendations for their indication and limitations. Evidence-based recommendations are essential for standardizing care quality and decision paths, despite some scientific evaluations showing contradictory assessments. Comparative studies on survival, quality, or caries susceptibility of different care types yield inconsistent results, underscoring the need for systematic review and evidence evaluation.

Part 1 of this guideline aims to present current evidence on the survival and quality of composite restorations in cavity Classes I–V and extended indication areas, such as direct posterior restorations with cusp replacement and direct tooth shape corrections in the anterior area.

This guideline is primarily aimed at all dentists. It is also intended to provide further information for patients and their caregivers.

## Methods

### General Framework

This guideline was formulated following the methodological standards set by the Standing Guideline Commission of the Association of Scientific Medical Societies in Germany (AWMF) (https://www.awmf.org/leitlinien/awmf-regelwerk/awmf-guidance.html) and the Grading of Recommendations Assessment, Development and Evaluation (GRADE) Working Group (https://www.gradeworkinggroup.org/). It was developed under the auspices of the German Society of Restorative Dentistry (Deutsche Gesellschaft für Zahnerhaltung, DGZ) and the German Society of Dentistry and Oral Medicine (Deutsche Gesellschaft für Zahn-, Mund- und Kieferheilkunde, DGZMK). To ensure comprehensive stakeholder representation, a guideline panel comprising dental professionals from 20 national societies/organizations (Table 1) was established. The development process was overseen by an Organizing Committee and a team of methodology consultants appointed by the DGZMK.

**Table 1:** Scientific societies/organizations represented in the guideline panel (in alphabetical order)

AGOKi	Working Group for Oral and Maxillofacial Surgery of the DGZMK (Arbeitsgemeinschaft für Oral- und Kieferchirurgie der DGZMK)
BuKiz	German Federal Association of Paediatric Dentists (Bundesverband der Kinderzahnärzte)
BZÄK	German Dental Association (Bundeszahnärztekammer)
BZÖG	German Federal Association of Dentists in the Public Health Service (Bundesverband der Zahnärzte des Öffentlichen Gesundheitsdienstes)
DEGUZ	German Society of Environmental Dentistry (Deutsche Gesellschaft für Umwelt-ZahnMedizin)
DGÄZ	German Association of Aesthetic Dentistry (Deutsche Gesellschaft für Ästhetische Zahnheilkunde)
DGCZ	German Society of Computer Aided Dentistry (Deutsche Gesellschaft für Computergestützte Zahnheilkunde)
DGET	German Association of Endodontics and Dental Traumatology (Deutsche Gesellschaft für Endodontologie und zahnärztliche Traumatologie)
DGKiZ	German Society of Paediatric Dentistry (Deutsche Gesellschaft für Kinderzahnheilkunde)
DGL	German Society of Laser Dentistry (Deutsche Gesellschaft für Laserzahnheilkunde)
DGoEV	German Society of Oral Epidemiology and Health Services Research (Deutsche Gesellschaft für Orale Epidemiologie und Versorgungsforschung)
DG Paro	German Society of Periodontology (Deutsche Gesellschaft für Parodontologie)
DGPro	German Society of Prosthetic Dentistry and Biomaterials (Deutsche Gesellschaft für Prothetische Zahnmedizin und Biomaterialien)
DGPZM	German Society of Preventive Dentistry (Deutsche Gesellschaft für Präventivzahnmedizin)
DGR2 Z	German Society of Restorative and Regenerative Dentistry (Deutsche Gesellschaft für Restaurative und Regenerative Zahnerhaltung)
DGZ	German Society of Restorative Dentistry (Deutsche Gesellschaft für Zahnerhaltung)
DNEBM	German Network of Evidence-based Medicine (Deutsches Netzwerk Evidenzbasierte Medizin)
FVDZ	Free Association of German Dentists (Freier Verband Deutscher Zahnärzte)
KZBV	German National Association of Statutory Health Insurance Dentists (Kassenzahnärztliche Bundesvereinigung)
VDZE	Association of German Certified Endodontists (Verband Deutscher Zertifizierter Endodontologen)

Participants in the guideline development were nominated, actively contributed to the process, and held voting rights during the consensus conference. The participants received guidance from the methodology consultants. However, these methodologists did not possess voting rights in the decision-making process.

### Key Questions – Definition of PICO

Key therapeutic questions were identified and reformulated as Population, Intervention, Comparator, and Outcome (PICO) questions.^41,49^ These were addressed in an evidence-based manner. Targeted patient population were patients with permanent tooth structure loss requiring restoration. This excludes patients with endodontically pre-treated teeth, those with build-up fillings, individuals affected by molar incisor hypomineralization or other structural anomalies, as well as those necessitating complete bite elevations.

The selection process, conducted by the guideline panel, prioritized clinical relevance and feasibility within the designated timeframe. The questions addressed are listed in Table 2.

**Table 2:** PICO(S) questions

PICO question	1	2	3	4	5
PICO aspect	Explanation				
Population	Patients with permanent teeth and carious defects requiring treatment, insufficient restorations or trauma (without endodontically pre-treated teeth, build-up fillings, MIH or other structural anomalies, bite elevations, pulp involvement, adhesion of tooth fragments)	Patients with permanent teeth and carious defects requiring treatment or insufficient restorations or trauma (without endodontically pre-treated teeth, build-up fillings, MIH or other structural anomalies, bite elevations)	Patients with permanent teeth and carious defects requiring treatment, insufficient restorations or trauma (without endodontically pre-treated teeth, build-up fillings, MIH or other structural anomalies, bite elevations)	Patients with permanent teeth and carious defects requiring treatment, insufficient restorations, trauma (without endodontically pre-treated teeth, build-up fillings, MIH or other structural anomalies, bite elevations) or the need for esthetic or functional corrections	Patients with permanent teeth and carious defects requiring treatment, insufficient restorations or hypersensitive teeth (without endodontically pre-treated teeth, build-up fillings, MIH or other structural anomalies)
Intervention	Direct composite restoration Class I and II	Extended direct composite restoration with cusp replacement	Direct composite restoration Class III and IV	Direct composite restoration, tooth shape correction	Direct composite restoration Class V
Comparison control	Direct restorations other than composite restorations Inlays; without partial crowns (limited, see below) The following applies: Posterior region: exclude partial crowns that replace all cusps, if not all cusps are replaced: include	Direct restorations other than composite restorations, Inlays, partial crowns (limited, see below) The following applies: Posterior region: exclude partial crowns that replace all cusps, if not all cusps are replaced: include	Search without specifying comparison, selection during screening Include veneers (cave: veneers only for the same indication, do not include purely esthetic veneers) Exclusion: partial crowns, full crowns	Crowns, partial crowns, veneers, selection of studies with comparable indications	Direct restorations other than composite restorations Non-invasive treatment
Outcome	Survival rate Failure analysis	Survival rate Failure analysis	Survival rate Failure analysis	Survival rate Failure analysis	Survival rate Failure analysis
Study type/setting	Study designs: systematic reviews, meta-analyses At least 12 months’ follow-up At least 15 restorations Publication since 1990 Languages: German, English, French, Russian	Study designs: CCTs, RCTs Systematic reviews, meta-analyses Prospective/retrospective cohort studies At least 12 months’ follow-up At least 15 restorations Publication since 1990 Languages: German, English, French, Russian	Study designs: CCTs, RCTs Systematic reviews, meta-analyses At least 12 months’ follow-up At least 15 restorations Publication since 1990 Languages: German, English, French, Russian	Study designs: CCTs, RCTs Systematic reviews, meta-analyses Prospective/retrospective cohort studies At least 12 months’ follow-up At least 15 restorations Publication since 1990 Languages: German, English, French, Russian	Study designs: Systematic reviews, meta-analyses At least 12 months’ follow-up At least 15 restorations Publication since 1990 Languages: German, English, French, Russian

CCT= controlled clinical trial, RCT = randomized clinical trial.

### Systematic Search Strategy

Two electronic databases, the National Library of Medicine, Washington, DC (MEDLINE via OVID) and the Cochrane Library (CENTRAL), were utilized for a comprehensive search addressing the research questions. Additionally, the reference lists of relevant manuscripts were manually reviewed. This systematic search, conducted up to December 2021, was performed independently by two investigators (CS and EL). Details of the search strategies for the PICO questions are shown in Table A.1 in the Appendix. The general inclusion criteria comprised studies with a follow-up period of at least 12 months, at least 15 restorations examined and publications from 1990 onwards that were published in English, German, French or Russian. The details of the included populations and study designs varied depending on the PICO question and can be found in the detailed table of PICO questions (Table 2). Studies that did not fulfill all inclusion criteria were excluded.

### Quality Assessment of Included Studies

The critical appraisal of evidence for PICO questions 1–5 was conducted by two independent investigators (CS and EK). The underlying evidence for the recommendations was systematically evaluated at the study or meta-analysis level, depending on the type of study selected. For randomized studies, the Cochrane Risk of Bias 2.0 (RoB 2) tool was employed,^58^ and for non-randomized studies, the ROBINS-I tool (Risk of Bias in Non-randomized Studies of Interventions) was used.^57^ Both tools include an endpoint-based assessment of the risk of bias. Systematic reviews were appraised using the AMSTAR 2 tool.^54^ The outcomes of these assessments, along with patient characteristics and study results, were compiled in evidence tables.

In those cases where comparators were available, the internationally recognized GRADE system25 (Grading of Recommendations Assessment, Development and Evaluation) was used to determine the confidence in the evidence. The GRADE system is an approach that assesses the certainty or confidence in the identified effect estimates of the included studies in relation to the selected outcomes. The evidence grading is divided into four levels (Table 3). These GRADE evaluations provided a foundation for balancing benefits and harms in formulating recommendations, with evaluations of primary outcomes and comparators detailed in Summary of Evidence tables. All evidence tables and GRADE Summary of Evidence tables are available in the evidence report from the AWMF website (https://register.awmf.org/de/leitlinien/detail/083-028). In cases where there were insufficient studies with comparators to apply the GRADE system, the Oxford Centre for Evidence-Based Medicine (OCEBM, https://www.cebm.ox.ac.uk/resources/levels-of-evidence/explanation-of-the-2011-ocebm-levels-of-evidence) level of evidence was used instead.

**Table 3:** Evidence grading (according to GRADE^25^)

Evidence	Description	Icon
High	We are very confident that the true effect is close to that of the estimate of the effect	⊕ ⊕ ⊕ ⊕
Moderate	We are moderately confident in the effect estimate: the true effect is likely to be close to the estimate of the effect, but there is a possibility that it is substantially different	⊕ ⊕ ⊕ ⊖
Low	Our confidence in the effect estimate is limited: The true effect may be substantially different from the estimate of the effect	⊕ ⊕ ⊖ ⊖
Very low	We have very little confidence in the effect estimate: the true effect is likely to be substantially different from the estimate of the effect	⊕ ⊖ ⊖ ⊖

### Formulation and Graduation of Recommendations and Structured Consensus Building

The comprehensive evidence report, including the systematic literature search and evidence tables for the respective PICO questions, was made available to the guideline panel members from February 13, 2022. This report was presented to the group on January 5, 2023. The guideline’s recommendations were then formulated in alignment with AWMF specifications. This process was conducted in separate working groups. Developed recommendations were discussed, debated if necessary, and approved in separate video conferences by each working group. In September 2023, these recommendations were consolidated into a master document and shared with the entire guideline panel. The voting on the recommendations occurred during the guideline consensus conference on November 7, 2023, in Heidelberg, moderated neutrally by the AWMF.

During the structured consensus conference (NIH type 1), the recommendations were agreed upon according to the following steps^20^:

1. Presentation of each recommendation or statement by the working group, with a brief explanation.
2. Reflection time for considering recommendation level, formulation, and alternatives, opportunity for queries and submission of reasoned amendments.
3. Preliminary voting, if necessary, to discuss individual comments and create a ranking.
4. Discussion of the points under debate.
5. Final voting on each recommendation and alternatives.
6. Repetition of these steps for each recommendation.

After editorial finalization, the updated guideline was reviewed and endorsed by the participating and leading societies/organizations. Tables 4 and 5 illustrate the applied scheme for determining the strength of the recommendations and the classification of consensus strength.

**Table 4:** Strength of recommendations: grading scheme (German Association of the Scientific Medical Societies [AWMF] and Standing Guidelines Commission)^10^

	Recommendation	Recommendation against intervention	Description	Symbol
A	Shall/We recommend	Shall not/We do not recommend	Strong recommendation	↑↑ resp. ↓↓
B	Should/We propose	Should not/We do not suggest	Recommendation	↑ resp. ↓
0	Can/May be considered	Can be dispensed with	Open recommendation	⇔

**Table 5:** Strength of consensus: determination scheme (German Association of the Scientific Medical Societies [AWMF] and Standing Guidelines Commission)^10^

Strong consensus	Agreement of >95% of participants
Consensus	Agreement of >75 to 95% of participants
Simple majority	Agreement of >50 to 75% of participants
No consensus	Agreement of <50% of the participants

## Results

**Fig 1:**
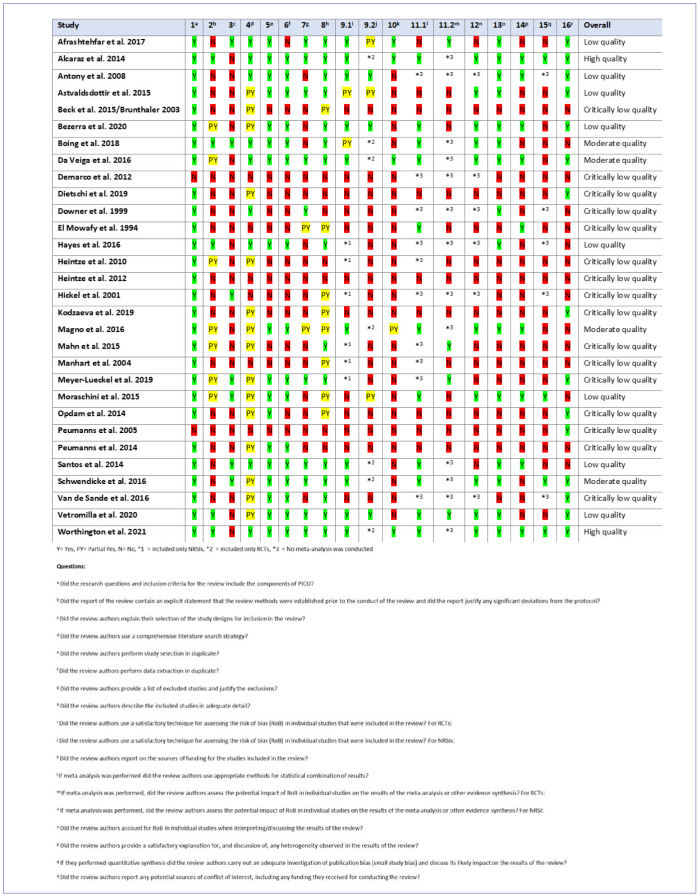
AMSTAR-2 evaluation of systematic reviews

**Fig 2:**
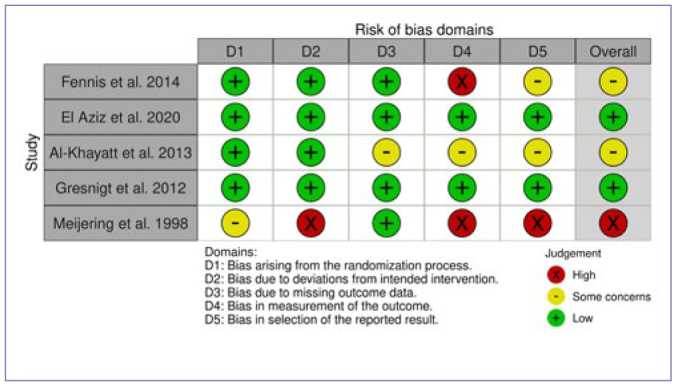
ROB 2 evaluation of randomized controlled clinical trials

**Fig 3:**
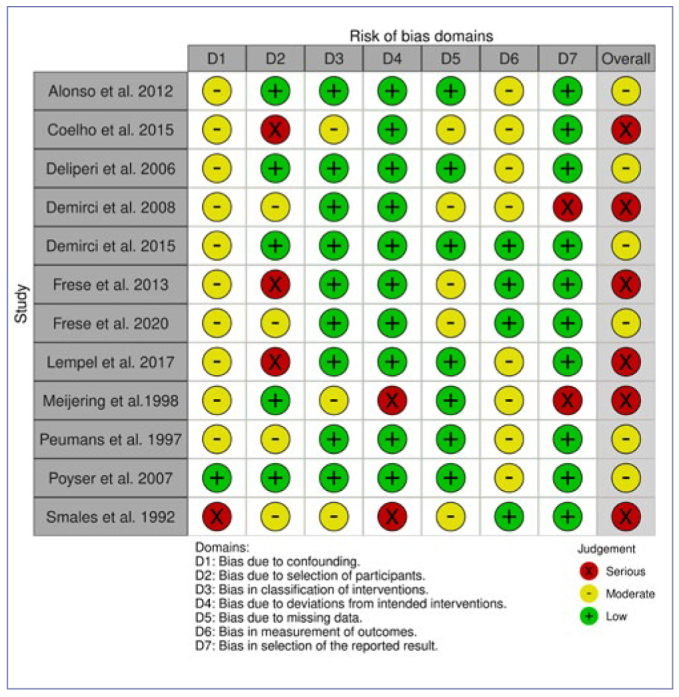
ROBINS-I evaluation of non-randomized clinical trials

**Fig A1:**
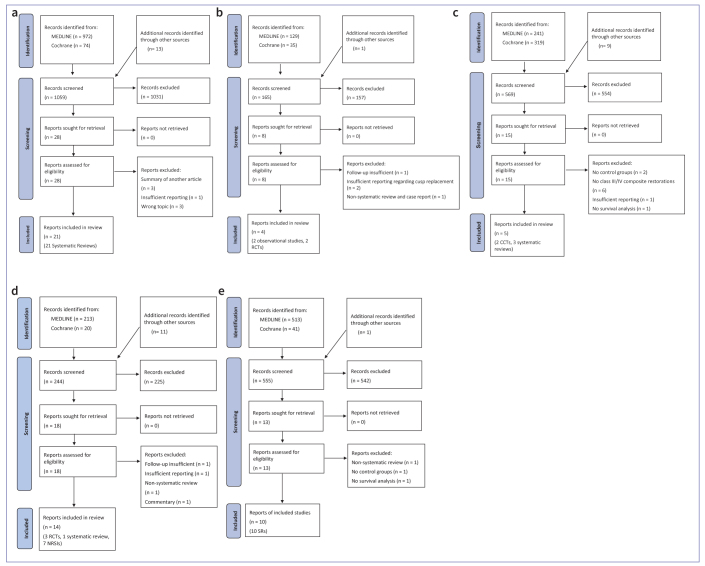
PRISMA Flow diagrams for the PICO questions. (a) PICO question #1, (b) PICO question #2, (c) PICO question #3, (d) PICO question #4, (e) PICO question #5.

PRISMA (Preferred Reporting Items for Systematic Reviews and Meta-Analyses) flow diagrams for literature selection, and comprehensive lists of excluded manuscripts with justifications for each PICO question are available in the Appendix (Fig A.1a–e, Table A.2). An overview of the AMSTAR 2, ROBINS-I, and RoB 2 assessments, depending on the study type, for all included studies, is illustrated in Figures 1–3.

Overall, all recommendations/statements were adopted by strong consensus. In total, part 1 of this guideline resulted in nine evidence-based recommendations and four consensus-based recommendations on indications for composite restorations (Tables 6–18).

### Direct Composite Restorations in Restoration Classes I and II

**Table 6:** Evidence-based recommendation 1

Composite restorations **can** be used for the direct restoration of Class I and II cavities. Vote: 17/0/0 (yes, no, abstention)		Strong consensus
Literature: Afrashtehfar et al., 2017,^1^ Rasines-Alcaraz et al., 2014,^48^ Antony et al., 2008,^4^ Downer et al., 1999,^17^ Heintze et al., 2012,^29^ Hickel et al., 2001,^32^ Manhart et al., 2004,^37^ Moraschini et al., 2015,^40^ Van de Sande et al., 2016,^59^ Vetromilla et al., 2020,^62^ Worthington et al. 2021,^65^		
Evidence base	11 systematic reviews (9 meta-analyses and 2 narrative reviews)	
Degree of recommendation	**0** ⇔	
Quality of the evidence	*Survival rate* Composite vs amalgam Composite vs glass-ionomer cement Composite vs ceramic *Secondary caries* Composite vs amalgam *Fracture* Composite vs amalgam	⊕ ⊕ ◯ ◯ (low) ⊕ ⊕ ◯ ◯ ◯ (very low) ⊕ ⊕ ◯ ◯ (low) ⊕ ⊕ ◯ ◯ (low) ⊕ ⊕ ◯ ◯ (low)

**Table 7:** Consensus-based recommendation 2

As an alternative to composite, glass-ionomer cement* **can** be used in specific indications (eg, smaller cavity sizes, limited compliance, increased caries risk) for the direct restoration of Class I and II cavities in permanent teeth. Vote: 17/0/0 (yes, no, abstention)	Strong consensus
Further reading: Vetromilla et al., 2020,^62^ Hickel et al., 2001,^32^ Manhart et al., 2004,^37^ Downer et al., 1999,^17^ Gurgan et al., 2020,^24^ Heck et al., 2020,^28^ Schwendicke et al., 2021,^53^ Rożniatowski et al., 2021,^50^ Wafaie et al., 2023^63^	

* This refers to glass-ionomer cements that are approved by the manufacturer for permanent use in the posterior region.

**Table 8:** Evidence-based recommendation 3

Indirect composite inlays **should not** be used for Class I and II cavities if they can be restored with direct composite restorations. Vote: 16/0/0 (yes, no, abstention)		Strong consensus
Literature: Da Veiga et al., 2016,^11^ Vetromilla et al., 2020,^62^ Hickel et al., 2001,^32^ Manhart et al., 2004^37^		
Evidence base	4 systematic reviews	
Degree of recommendation	B ⇓	
Quality of the evidence	*Survival rate* Direct composite restoration vs. indirect composite restoration	⊕ ⊕ ⊕ ◯ (moderate)

**Table 9:** Evidence-based recommendation 4

If Class I and II cavities cannot be restored with direct composite restorations, indirect ceramic restorations or cast metal restorations **can** be used as an alternative. Vote: 16/0/0 (yes, no, abstention)		Strong consensus
Literature: Hickel et al., 2001,^32^ Manhart et al., 2004^37^		
Evidence base	2 systematic reviews	
Degree of recommendation	**0** ⇔	
Quality of the evidence	*Survival rate* Composite vs ceramic	⊕ ⊕ ◯ ◯ (low)

### Direct Composite Restorations with Cusp Replacement in Posterior Restorations

**Table 10:** Evidence-based recommendation 5

Composite restorations **can** be used for cavities with cusp replacements in the posterior region. Vote: 16/0/0 (yes, no, abstention)		Strong consensus
Literature: Van Nieuwenhuysen et al., 2003,^61^ Deliperi et al., 2016^12^		
Evidence base	2 observational studies	
Degree of recommendation	**0** ⇔	
Quality of the evidence	Composite vs. amalgam Survival rate Secondary caries Fracture of the restoration Cusp fracture	⊕ ◯ ◯ ◯ ◯ (very low) ⊕ ◯ ◯ ◯ ◯ (very low) ⊕ ◯ ◯ ◯ ◯ (very low) ⊕ ◯ ◯ ◯ ◯ (very low)

**Table 11:** Consensus-based recommendation 6

Indirect composite restorations **can** be used for cavities with cusp replacement in the posterior region, especially when there are specific tooth, mouth or patient factors (e.g. limited compliance, poor accessibility, complex functional rehabilitation, etc.). Vote: 16/0/1 (yes, no, abstention)	Strong consensus
Further reading: El Aziz et al., 2020,^18^ Fennis et al., 2014^19^	

### Direct Composite Restorations in Restoration Classes III and IV

**Table 12:** Evidence-based recommendation 7

Direct composite materials **shall** be used to restore Class III and IV defects. Vote: 17/0/0 (yes, no, abstention)		Strong consensus
Literature: Demarco et al., 2015,^13^ Demirci et al., 2008,^15^ Dietschi et al., 2019,^16^ Heintze et al., 2015,^30^ Smales et al., 1992^55^		
Evidence base	3 systematic reviews, 2 controlled clinical studies	
Degree of recommendation	A ⇑⇑	
Level of evidence	Level 2	

**Table 13:** Consensus-based recommendation 8

Glass-ionomer cements **should not** be used for the permanent restoration of Class III and IV defects. Vote: 15/0/1 (yes, no, abstention)	Strong consensus
Further literature: Heintze et al. 2015^30^	

### Direct Composite Restorations for Tooth Shape Corrections in the Anterior Region

**Table 14:** Evidence-based recommendation 9

Direct composite materials **shall** be used for tooth shape corrections in the anterior region. Vote: 16/0/0 (yes, no, abstention)		Strong consensus
Literature: Alonso et al., 2012,^3^ Poyser at al., 2007,^47^ Al Khayatt et al., 2013,^2^ Coelho et al., 2015,^8^ Demarco et al., 2015,^13^ Demirci et al., 2015,^15^ Frese et al., 2013,^21^ Frese et al., 2020,^22^ Wolff et al., 2010,^64^ Gresnigt et al., 2012,^23^ Lempel et al., 2017,^35^ Meijering et al., 1998,^38^ Peumans et al., 1997,^46^ Peumans et al., 1997^45^		
Evidence base	1 systematic review, 3 randomized controlled clinical trials 10 non-randomized studies	
Degree of recommendation	A ⇑⇑	
Level of evidence	Level 2	

**Table 15:** Consensus-based recommendation 10

For tooth shape correction in the anterior region, minimally invasive direct procedures that preserve tooth structure **shall** be preferred whenever possible; indirect ceramic veneers can be used as an alternative. Vote: 16/0/0 (yes, no, abstention)	Strong consensus
Further reading: Meijering et al., 1998^38^	

### Direct Composite Restorations in Restoration Class V

**Table 16:** Evidence-based recommendation 11

For Class V restorations, direct composite materials **can** be used if adequate contamination control and adhesive technique are ensured. Vote: 16/0/0 (yes, no, abstention)		Strong consensus
Literature: Bezerra et al., 2020,^5^ Boing et al., 2018,^6^ Hayes et al., 2016,^27^ Heintze et al., 2010,^31^ Mahn et al., 2015,^36^ Meyer-Lückel et al., 2019,^39^ Peumans et al., 2005,^44^ Peumans et al., 2014,^43^ Santos et al., 2014,^51^ Schwendicke et al., 2016^52^		
Evidence base	10 systematic reviews	
Degree of recommendation	**0** ⇔	
Quality of the evidence	Composite vs glass-ionomer cement Retention Marginal adaptation	⊕ ⊕ ◯ ◯ (low) ⊕ ⊕ ◯ ◯ (low)

**Table 17:** Evidence-based recommendation 12

As an alternative to composite, glass-ionomer cements/modified glass-ionomer cements** can** be used to restore Class V defects. Vote: 16/0/0 (yes, no, abstention)		Strong consensus
Literature: Bezerra et al., 2020,^5^ Boing et al., 2018,^6^ Hayes et al., 2016,^27^ Heintze et al., 2010,^31^ Mahn et al., 2015,^36^ Meyer-Lückel et al., 2019,^39^, Peumans et al., 2005,^44^ Peumans et al., 2014,^43^ Santos et al., 2014,^51^ Schwendicke et al., 2016^52^		
Evidence base	10 systematic reviews	
Degree of recommendation	**0** ⇔	
Quality of the evidence	Composite vs glass-ionomer cement Retention Marginal adaptation	⊕ ⊕ ◯ ◯ (low) ⊕ ⊕ ◯ ◯ (low)

**Table 18:** Evidence-based recommendation 13

If direct composite restorations are used to restore Class V defects, 2-step-self-etch, 3-step-etch-and-rinse adhesive systems or universal adhesives **should** be used. Vote: 16/0/0 (yes, no, abstention)		Strong consensus
Literature: Heintze et al., 2010,^31^ Mahn et al., 2015,^36^ Meyer-Lückel et al., 2019,^39^ Peumans et al., 201443		
Evidence base	4 systematic reviews	
Degree of recommendation	B ⇑	
Level of evidence	Level 2	

## Discussion

To the best of our knowledge, this S3 clinical practice guideline is the first of its kind based on a systematic literature review, an assessment of the quality of evidence, and the use of formal consensus methods. In the context of Class I and II cavities, the assessed reviews indicate a higher likelihood of restoration loss and secondary caries with composite restorations compared to amalgam restorations, as shown by the effect estimates for survival rates. However, for fractures, there was no significant difference in occurrence between composite and amalgam restorations.

The confidence in these effect estimates is, nonetheless, limited. The clinical significance of these findings is constrained by many studies focusing primarily on children,^48, 40, 62, 65^ a group with potentially lower compliance and uncertain caries risk. Since amalgam usage is now outdated in this patient group, basing a recommendation for action solely on this data is inappropriate. Studies without a comparator reported satisfactory survival rates and acceptable annual failure rates for composites, especially when using a 2-step-self-etch or 3-step-etch-and-rinse technique.^52^ Two systematic reviews^32,37^ found no statistically significant differences between amalgam and composite restorations, suggesting that they might be clinically equivalent, though this excludes children and adolescents. Patient-specific risk factors, particularly caries risk, significantly affect the survival of composite restorations and should be considered in clinical decision-making and interpretation of study data.^14,29,42,60,62^ The decision to recommend composites for Class I and II restorations is based on the assessment of the equivalence of the two restoration types, which can be derived from the synthesis of the effect estimates and the further clinical data.^32,37,40,48,62,65^ Direct composite restorations are better than amalgam restorations in terms of minimally invasive dentistry. In patient groups with a high caries risk, however, amalgam or glass-ionomer restorations may be advantageous. The comparison of effect estimates between direct and indirect composite restorations indicates equivalent or lower survival for indirect composite restorations, with a moderate level of confidence in this estimate. It is judged that the actual effect is likely close to this estimate. Based on this, the evidence-based recommendation is to prefer direct composite restorations over indirect ones in Class I and II cavities. Besides the anticipated marginally better survival rates, the significant advantage of greater tooth structure preservation is especially relevant.

Regarding the evidence on composite use for posterior cavities with cusp replacement, a number of studies showed acceptable failure rates.^12,18,19^ In contrast, Van Nieuwenhuysen et al^61^ observed high failure rates for composite and amalgam restorations in this type of restoration (30.4% and 28.1%, respectively), but these data are considered outdated due to the use of older composite materials. Modern composites are likely to perform better, aligning with minimally invasive dentistry principles by preserving tooth structure and offering better fracture resistance.

For Class III and IV restorations, the evidence from the studies included in this analysis shows high survival rates for composite restorations in these classes.^13,15,16,30,55^ A notable aspect of this evidence is the relatively short follow-up period, often only 2 years, while follow-ups exceeding 10 years for such restorations are rare. This suggests that longer-term studies might reveal different outcomes. The studies also compared composite materials with varying filler sizes and matrix compositions. Here, microfiller composites or those with smaller particle sizes showed lower survival rates compared to hybrid composites. However, it is important to consider the evolution of these materials into today’s nano-filled composites, which likely have improved survival rates. In studies using comparators, no substantial differences were noted between composite and compomer restorations, although these also had relatively short follow-up periods. However, for Class III restorations, composites demonstrated better wear resistance and anatomical stability compared to glass-ionomer cements. The overall benefit-harm assessment for using composites in the restoration of Class III and IV defects strongly supports their use. This is due to the high survival rates and good to excellent clinical quality of the restorations. Additionally, adhesive restorations are preferred over retentively anchored or indirect alternatives, considering their lower invasiveness.

Similar results were observed regarding tooth shape corrections. The studies reviewed demonstrated high to very high survival rates for tooth shape corrections using composites, lasting up to 15 years. No significant differences were observed between different material groups in terms of restoration survival, suggesting that longer follow-up periods may not yield significant changes in outcomes. Among the various composite materials, microfiller composites showed better esthetic outcomes compared to universal composites, though one study noted higher discoloration with nano-filled composites. A higher incidence of fractures in tooth shape corrections was reported with microhybrid composites in one study. When comparing composite with indirect ceramic veneers, the latter showed significantly higher survival rates, although this conclusion is based on just one study with a relatively short follow-up period.^38^ The benefit-harm assessment for using composites for tooth shape correction in the anterior region strongly favors their use, considering their high survival rates, excellent to good clinical quality, the repairability of composites, and less invasiveness compared to traditional ceramic veneers. The elective nature of these procedures should be considered in the overall assessment. A minimally or non-invasive and prevention-oriented approach is recommended for these treatments.

Direct Class V composite restorations demonstrated high survival rates and low annual failure rates over long-term observations (12 months to 13 years).^5,6,27,31,36,39,43,44,51,52^ Each of these reviews included at least one comparator, with glass-ionomer cements or modified glass-ionomer cements being commonly used across studies. However, only limited evidence was found for compomers and ormocers,^52^ resulting in no specific recommendation for their use. The retention of Class V restorations emerged as a primary focus, with glass-ionomer cements or modified glass-ionomer cements generally outperforming other materials. Nevertheless, the adhesive protocol played an essential role in the retention of composites in Class V cavities. Specifically, 3-step-etch-and-rinse, 2-step-self-etch, and universal adhesives were crucial in achieving long-term retention comparable to that of glass-ionomer cements or modified glass-ionomer cements. In the case of other clinical quality parameters such as marginal adaptation, anatomical shape, surface texture and condition, and secondary caries, composites performed similar to glass-ionomer cements or modified glass-ionomer cements.

The guideline is the first to provide comprehensive evidence on the use of direct composite materials. In conclusion, this guideline recommends the use of composite materials for direct restoration of Class I and II cavities, supported by strong expert consensus and a broad evidence base. Glass-ionomer cements are acknowledged as alternative materials for specific situations, such as smaller cavities or higher caries risk in these cavity classes. The use of indirect composite inlays is discouraged in favor of direct restorations when feasible, and direct composites are specifically recommended for Class III and IV defects. The guideline also recommends composite restorations for posterior cavities requiring cusp replacements and, in some instances, indirect composites. For anterior tooth shape correction, direct composite restorations are preferred and recommended, because they are particularly suitable for minimally invasive and prevention-oriented treatment concepts. However, it is clear that, particularly in the area of posterior restorations and Class V, the evidence base should be expanded in the future with long-term clinical studies in comparison to the comparators amalgam, (modified) glass-ionomer cements and due to current legislation on the amalgam phase-out, also with amalgam replacement materials. Regular updates of this guideline can therefore highlight future areas and limitations of direct composite restorations in detail.

### Clinical Relevance Statement

This guideline provides evidence-based recommendations for using composite materials in direct restorations of permanent teeth, outlining appropriate indication areas.

## Appendix Part 1

**Table A1:** MEDLINE search term via OVID for the PICO questions

PICO question #1	PICO question #2	PICO question #3	PICO question #4	PICO question #5
dentition, permanent/or exp tooth/ permanent Dentition.mp. permanent teeth.mp. secondary Dentition.mp. secondary teeth.mp. adult teeth.mp. adult tooth.mp. permanent tooth.mp. secondary tooth.mp. adult Dentition.mp. 1 or 2 or 3 or 4 or 5 or 6 or 7 or 8 or 9 or 10 exp Tooth Diseases/ exp Dental Caries/ caries.mp. dental caries.mp. carious lesion*.mp. tooth Decay.mp. dental Cavit*.mp. Cavit*.mp. demineralization*.mp. dental Trauma.mp. 12 or 13 or 14 or 15 or 16 or 17 or 18 or 19 or 20 or 21 exp bicuspid/ or exp molar/ molar*.mp. bicusp*.mp. premolar*.mp. posterior teeth.mp. posterior tooth.mp. class I.mp. class II.mp. 23 or 24 or 25 or 26 or 27 or 28 or 29 or 30 exp dental restoration failure/ or exp dental restoration, permanent/ or exp dental restoration repair/ or dental marginal adaptation/ or exp diagnosis, oral/ exp Composite Resins/ dental restoration*.mp. filling*.mp. restoration*.mp. composit*.mp. 32 or 33 or 34 or 35 or 36 or 37 Randomized Controlled Trials as Topic/ exp Controlled Clinical Trial/ RCT*.mp. randomized controlled Trial*.mp. randomised controlled Trial*.mp. systematic review*.mp. meta Analysis.mp. controlled clinical Trial.mp. randomized.mp. randomised.mp. controlled clinical Trial*.mp. cct*.mp. 39 or 40 or 41 or 42 or 43 or 44 or 45 or 46 or 47 or 48 or 49 or 50 11 and 22 and 31 and 38 and 51 limit 52 to (yr=”1990 -Current” and (english or french or german or russian))	dentition, permanent/ or exp tooth/ permanent Dentition.mp. permanent teeth.mp. secondary Dentition.mp. secondary teeth.mp. adult teeth.mp. adult tooth.mp. permanent tooth.mp. secondary tooth.mp. adult Dentition.mp. 1 or 2 or 3 or 4 or 5 or 6 or 7 or 8 or 9 or 10 exp Tooth Diseases/ exp Dental Caries/ caries.mp. dental caries.mp. carious lesion*.mp. tooth Decay.mp. dental Cavit*.mp. Cavit*.mp. demineralization*.mp. dental Trauma.mp. 12 or 13 or 14 or 15 or 16 or 17 or 18 or 19 or 20 or 21 exp bicuspid/ or exp molar/ molar*.mp. bicusp*.mp. premolar*.mp. posterior teeth.mp. posterior tooth.mp. 23 or 24 or 25 or 26 or 27 or 28 exp dental restoration failure/ or exp dental restoration, permanent/ or exp dental restoration repair/ or dental marginal adaptation/ or exp diagnosis, oral/ exp Composite Resins/ dental restoration*.mp. filling*.mp. restoration*.mp. composit*.mp. 30 or 31 or 32 or 33 or 34 or 35 cusp replac*.mp. cuspal restoration*.mp. cuspal Coverage*.mp. cusp-replac*.mp. onlay.mp. 37 or 38 or 39 or 40 or 41 11 and 22 and 29 and 36 and 42 limit 43 to (yr=”1990 -Current” and (english or french or german or russian))	dentition, permanent/ or exp tooth/ permanent Dentition.mp. permanent teeth.mp. secondary Dentition.mp. secondary teeth.mp. adult teeth.mp. adult tooth.mp. permanent tooth.mp. secondary tooth.mp. adult Dentition.mp. 1 or 2 or 3 or 4 or 5 or 6 or 7 or 8 or 9 or 10 exp Tooth Diseases/ exp Dental Caries/ caries.mp. dental caries.mp. carious lesion*.mp. dental Cavit*.mp. Cavit*.mp. demineralization*.mp. dental Trauma.mp. tooth Decay.mp. 12 or 13 or 14 or 15 or 16 or 17 or 18 or 19 or 20 or 21 exp dental restoration failure/ or exp dental restoration, permanent/ or exp dental restoration repair/ or dental marginal adaptation/ or exp diagnosis, oral/ dental restoration*.mp. exp Composite Resins/ filling*.mp. restoration*.mp. composit*.mp. 23 or 24 or 25 or 26 or 27 or 28 exp cuspid/ or exp incisor/ anterior tooth.mp. anterior teeth.mp. anterior*.mp. front* teeth.mp. front* tooth.mp. front*.mp. incisor*.mp. cuspid*.mp. canine*.mp. class III.mp. class IV.mp. 30 or 31 or 32 or 33 or 34 or 35 or 36 or 37 or 38 or 39 or 40 or 41 Randomized Controlled Trials as Topic/ exp Controlled Clinical Trial/ RCT*.mp. randomized controlled Trial*.mp. randomised controlled Trial*.mp. systematic review*.mp. meta Analysis.mp. controlled clinical Trial.mp. randomized.mp. randomised.mp. controlled clinical Trial*.mp. cct*.mp. 43 or 44 or 45 or 46 or 47 or 48 or 49 or 50 or 51 or 52 or 53 or 54 11 and 22 and 29 and 42 and 55 limit 56 to (yr=”1990 -Current” and (english or french or german or russian))	dentition, permanent/ or exp tooth/ permanent Dentition.mp. permanent teeth.mp. secondary Dentition.mp. secondary teeth.mp. adult teeth.mp. adult tooth.mp. permanent tooth.mp. secondary tooth.mp. adult Dentition.mp. 1 or 2 or 3 or 4 or 5 or 6 or 7 or 8 or 9 or 10 exp dental restoration failure/ or exp dental restoration, permanent/ or exp dental restoration repair/ or dental marginal adaptation/ or exp diagnosis, oral/ exp Composite Resins/ dental restoration*.mp. filling*.mp. restoration*.mp. composit*.mp. 12 or 13 or 14 or 15 or 16 or 17 exp cuspid/ or exp incisor/ anterior tooth.mp. anterior teeth.mp. anterior*.mp. front* teeth.mp. front* tooth.mp. front*.mp. incisor*.mp. cuspid*.mp. canine*.mp. anterior*.mp. 19 or 20 or 21 or 22 or 23 or 24 or 25 or 26 or 27 or 28 or 29 Composite buildup*.mp. recontour*.mp. Diastema*.mp. Composite veneer*.mp. shape correction*.mp. 31 or 32 or 33 or 34 or 35 11 and 18 and 30 and 36 limit 37 to (yr=”1990 -Current” and (english or french or german or russian)	exp Tooth Diseases/ exp Dental Caries/ exp Dentin Sensitivity/ exp Tooth Wear/ caries.mp. defect*.mp. lesion*.mp. carious.mp. non-carious.mp. dental Cavit*.mp. Cavit*.mp. demineralization*.mp. dental Trauma.mp. tooth Decay.mp. dent* hypersensitivity.mp. hypersensitiv*.mp. 1 or 2 or 3 or 4 or 5 or 6 or 7 or 8 or 9 or 10 or 11 or 12 or 13 or 14 or 15 or 16 cervical.mp. cervical lesion*.mp. wedge-shaped.mp. class V.mp. 18 or 19 or 20 or 21 exp dental restoration failure/ or exp dental restoration, permanent/ or exp dental restoration repair/ or dental marginal adaptation/ or exp diagnosis, oral/ exp Composite Resins/ dental restoration*.mp. filling*.mp. restoration*.mp. composit*.mp. 23 or 24 or 25 or 26 or 27 or 28 Randomized Controlled Trials as Topic/ exp Controlled Clinical Trial/ RCT*.mp. randomized controlled Trial*.mp. randomised controlled Trial*.mp. systematic review*.mp. meta Analysis.mp. controlled clinical Trial.mp. randomized.mp. randomised.mp. controlled clinical Trial*.mp. cct*.mp. 30 or 31 or 32 or 33 or 34 or 35 or 36 or 37 or 38 or 39 or 40 or 41 17 and 22 and 29 and 42 limit 43 to (yr=”1990 -Current” and (english or french or german or russian))

**Table A2:** Excluded publications with reasons

PICO question	Publication	Reason for exclusion
1	Balevi 2014^5^	Summary of a partial aspect of the study by Opdam et al., 2014.
	Farsai 2017^9^	Summary of the article by Da Veiga et al., 2016
	Frencken 2021^10^	Results for composite and amalgam vs GIZ not reported separately
	Fron Chabouis 2013^11^	Wrong topic, only indirect methods compared
	Hurst 2014^13^	Summary of the article by Alcaraz et al., 2014
	Kielbassa 2015^14^	Wrong topic
	Thighs 2019^22^	Wrong topic
2	Behle 1997^6^	Non-systematic review and case report
	Kujis 2006^15^	Follow-up insufficient
	Schwendicke 2016^24^	Insufficient reporting regarding cusp replacement
	Van Dijken 2000^25^	Insufficient reporting regarding cusp replacement
3	Al Khayatt 2013^2^	No Class III/IV composite restorations
	Antony 2008^3^	No Class III/IV composite restorations
	Baillod 1994^4^	No other material in the control group (except GIZ liner)
	Helbig 2002^12^	No other material in the control group
	Meijering 1998^17^	No Class III/IV composite restorations
	Narhi 2003^18^	Insufficient reporting
	Prakki 2008^21^	No Class III/IV composite restorations
	Schwendicke 2015^23^	No survival analysis
	Schwendicke 2016^24^	No Class III/IV composite restorations
	Van dijken et al., 1999^26^	No Class III/IV composite restorations
4	Ajlouni 2006^1^	Commentary
	Belcheva 2001^7^	Follow-up insufficient
	Dostalova 2013^8^	No separate reporting of the composite restorations
	Mangani 20071^6^	Non-systematic review
5	de Paula 20191^9^	No other material as control group (except GIZ liner)
	Pecie 2011^20^	Non-systematic review
	Schwendicke 2015^23^	No survival analysis of Class V restorations
